# Mixed Consistencies in Dysphagic Patients: A Myth to Dispel

**DOI:** 10.1007/s00455-021-10255-x

**Published:** 2021-02-17

**Authors:** Mozzanica Francesco, Pizzorni Nicole, Scarponi Letizia, Bazzotti Claudia, Ginocchio Daniela, Schindler Antonio

**Affiliations:** 1 Department of Clinical Sciences and Community Health, University of Milan, Via San Vittore 12, Milan, Italy; 2 IRCCS Multimedica, Ospedale San Giuseppe, Milan, Italy; 3 Department of Biomedical and Clinical Sciences, L. Sacco Hospital, University of Milan, Milan, Italy; 4 IRCCS Santa Maria Nascente – Fondazione Don Gnocchi, Milan, Italy

**Keywords:** Deglutition, Dysphagia, Mixed consistencies, Deglutition disorders, FEES

## Abstract

Only limited and inconsistent information about the effect of mixed consistencies on swallowing are available. The aim of this study was to evaluate the location of the head of the bolus at the swallow onset, the risk of penetration/aspiration, and the severity of post-swallow pharyngeal residue in patients with dysphagia when consuming mixed consistencies. 20 dysphagic patients underwent a Fiberoptic Endoscopic Evaluation of Swallowing (FEES) testing five different textures: liquid, semisolid, solid, biscuits-with-milk and vegetable-soup. The location of the head of the bolus at the onset of swallowing was rated using a five-points scale ranging from zero (the bolus is behind the tongue) to four (the bolus falls into the laryngeal vestibule), the severity of penetration/aspiration was rated using the Penetration Aspiration Scale (PAS), the amount of pharyngeal residue after the swallow was rated using the Yale Pharyngeal Residue Severity Rating Scale (YPRSRS) in the vallecula and pyriform sinus. When consuming biscuits-with-milk and liquid the swallow onset occurred more often when the boluses were located in the laryngeal vestibule. Penetration was more frequent with biscuits-with-milk, while aspiration was more frequent with Liquid, followed by biscuits-with-milk and vegetable-soup, Semisolid and Solid. In particular, no differences in penetration and aspiration between liquids and biscuits-with-milk were found as well as among vegetable-soup, semisolid and solid. No significant differences in the amount of food residue after swallowing were demonstrated. The risk of penetration-aspiration for biscuits-with-milk and liquid is similar, while the risk of penetration-aspiration is lower for vegetable-soup than for liquid.

## Introduction

Fiberoptic Endoscopic Evaluation of Swallowing (FEES) and Videofluoroscopic Swallowing Study (VFSS) are the gold standards for the assessment of dysphagia [1, 2]. Compared with VFSS, FEES examination offers several benefits, such as no radiation exposure (allowing repeated examinations at frequent intervals) [3], minimal invasiveness [4], portability and inferior costs [5]. Most of the FEES protocols test different single consistencies, including liquid, semisolid, and solid [6]. However, it should be argued that this protocol does not fully reflect real-life swallowing since a regular diet usually includes mixed consistencies [7]. In particular, the combination of liquid and solid phases in a single food is common during an ordinary meal, as in the case of a vegetable soup and biscuits-with-milk (which represent the commonest consumed food during a typical Italian dinner and breakfast respectively [8]).

Previous studies [9–12] demonstrated that swallowing mixed foods appear to be quite different from swallowing single consistency foods. In single consistency food, for example, liquids are generally held in the oral cavity until swallow onset, while solid foods are propelled into the oropharynx before swallow onset. When swallowing a mixed food, the liquid phase may reach the hypopharynx while the solid phase is still in the oropharynx. Humbert et al. [12] used VFSS to study swallowing kinematics and the location of the bolus at swallow onset with a variety of consistencies including two different mixed consistencies. They found that the healthy adults would regularly let the liquid portion of the mixed consistency fall to the pyriform sinuses while orally manipulating the more solid portion of the bolus [9]. Some authors speculated that this phenomenon might increase the risk of penetration-aspiration in dysphagic patients [9, 10] but there are inconsistencies in the international literature about this topic and only few studies have been reported so far. Two behaviors have been observed in managing a mixed consistency made of thin barium with chocolate chips: (1) swallow the liquid and then chew the solid part; (2) chew the solid part and then swallow the whole mixed bolus [12]. During solid food oral preparation, premature loss of the chewed bolus to the valleculae during mastication is known to be normal, because the soft palate seal to the back of the tongue is broken by active tongue movement during the chewing [13]; in case of mixed consistencies the lack of contact between tongue and soft palate might contribute to the passage of liquids to the pyriform sinuses. Ozaki et al. [10] studied through VFSS the risk of penetration-aspiration with different consistencies (pudding thick barium, thin liquid barium, corned beef hash with barium, a mix of corned beef hash with barium and thin liquid barium) in a group of 229 patients with suspected dysphagia of different origins; they reported that the mixed consistency (corned beef hash mixed with thin liquid barium) had the highest risk of aspiration for patients with dysphagia, while drinking thin liquid barium from a cup had the highest risk of penetration. More recently, Lee et al. [7] studied a group of 29 patients with dysphagia due to different neurological diseases using VFSS; thin liquid barium, rice, and a mix of both rice and thin liquid barium were given and penetration-aspiration, location of the leading edge at the onset of pharyngeal swallow and residue were analysed. They reported that swallowing of mixed consistency is safer for not inducing penetration-aspiration as compared with the swallowing of liquid; no difference between thin liquid and mixed consistencies in the location of the leading edge at the onset of pharyngeal swallow was found, but residue in the valleculae was greater with the mixed consistency compared with thin liquid. Kang et al. [14] analysed the swallow characteristics of a mixed consistency (thin liquid barium and steamed rice) during VFSS in a group of 49 patients after stroke or brain injuries who did not show overt dysphagia symptoms during bedside examination nor abnormal findings during VFSS with different single consistencies. In 24 patients (49%) abnormal findings with mixed consistency included spillage of liquids during oral preparation (23 patients), penetration (6 patients), aspiration (2 patients), and residue in the valleculae (2 patients) were found. Moreover, in these 24 patients pharyngeal transit time and initiation of the pharyngeal swallow observed to be significantly delayed as compared with the remaining 25 patients without abnormal findings with mixed consistency was found.

Swallowing of mixed consistencies in patients with dysphagia has been studied mainly through VFSS; therefore, the liquids were always represented by thin barium and no study analyzed swallowing of mixed consistencies through FEES using ordinary thin liquids and food meals. Moreover, swallowing of different types of mixed consistencies, including thicker and thinner mixed consistencies, has never been analyzed in patients with dysphagia. The aim of this study is to analyze the effect of a thinner and a thicker mixed consistency on swallow in patients with dysphagia using FEES. The underlying hypothesis is that the characteristics of mixed food affect the physiology of swallowing, and that the risk of penetration/aspiration, the severity of pharyngeal residue after swallowing and the location of the head of the bolus at the swallow onset with mixed consistencies are different from those with single consistencies in dysphagic patients. The importance of this study lies on the fact that an improved understanding of the impact of different mixed consistencies on swallowing could provide clinicians with a better understanding of patient’s swallowing abilities in functional situations and may support clinical decision making.

## Materials and Methods

The present study was conducted in accordance with the Declaration of Helsinki and it was previously approved by the Institutional Review Board of our hospital. All the data were collected prospectively.

### Participants

A total of 20 consecutive patients (7 females and 13 males) evaluated for swallowing disorders were recruited. All the enrolled subjects met the following inclusion criteria: age above 18 years; functional oral status (all natural teeth or partial tooth loss rehabilitated with an adjusted partial dental prosthesis [15]), full oral diet, Montreal Cognitive Assessment (MoCA) score of 26 or higher [16]; no medical history of gastroenterological, respiratory, rheumatologic, metabolic, hematologic, or neoplastic disorders. Exclusion criteria were: presence of oral pathologies which may affect mastication, intolerance to the components of the tested foods. The etiology of dysphagia was: stroke (4 patients), ischemic leukoariosis (8 patients), polyneuropathy (3 patients), amyotrophic lateral sclerosis (1 patient), Steinert’s disease (1 patients), systemic sclerosis (1 patient), ataxia (1 patient), Guillain-Barré syndrome (1 patient). All the patients received exhaustive explanations regarding aims and objectives of the research, FEES evaluation and all the possible risks involved. All the enrolled patients gave their written informed consent. Information regarding patient’s oral intake were collected using the Functional Oral Intake Scale (FOIS) [17]. This is a seven-point ordinal scale that describes the typical functional oral intake of patients and indicates limitations in oral intake of food and liquid; it ranges from one (nothing by mouth) to seven (total oral diet with no restriction). The FOIS was administered immediately before the FEES examination. The characteristics of the enrolled patients are reported in Table 1.

**Table 1:** Characteristics of the enrolled population

	Total
Sex	7 females 13 males
Age	74.5 years (70.25–80.5 years)
Weight	69 kg (60.5–73 kg)
FOIS	5 (5–6)
Etiology of dysphagia	
Stroke	4 patients
Ischemic leukoariosis	8 patients
Polyneuropathy	3 patient
ALS	1 patient
Steinert’s disease	1 patient
Systemic sclerosis	1 patient
Ataxia	1 patient
Guillain-Barré syndrome	1 patient

Medians are reported as well as interquartile ranges (in brackets)

*ALS* amyotrophic lateral sclerosis

### FEES Examination

Each FEES was conducted by the senior Phoniatrician. For this purpose, a XION EF-N flexible endoscope with a diameter of 3.4 mm and a length of 320 mm (XION GmbH, Berlin, Germany) mounted on an EndoSTROB E camera (XION GmbH, Berlin, Germany) were used. All the videos were processed using the software Daisy Viewer 2.0 (INVENTIS srl, Padua, Italy) and stored in an anonymous form in.AVI format.

Each subject was seated on a comfortable chair, leaning back (between 75° and 90° approximately) with his arms on the armrests and keeping the head in neutral position to obtain the best posture for the examination. No local anesthetic drugs (e.g. lidocaine spray) were used in order not to alter pharyngo-laryngeal sensibility. The endoscope was introduced into the widest nasal cavity and kept at a level just inferior to the uvula to maximize the field of view, including the larynx, the glossoepiglottic valleculae and the pyriform sinuses [18]. Five different textures of food, including two different mixed consistencies, were provided during FEES examination to evaluate swallowing:

1. Liquid: room temperature skim milk (< 50 mPa s at 50 and 300 s^−1^; International Dysphagia Diet Standardisation Iniatiative—IDDSI Level 0) [19] was used for thin Liquid trials. A volume of 10 mL was administered using a spoon
2. Semisolid: room temperature Crème Line vanilla pudding (Nutrisens Medical SAS, Francheville, France) (2583.3 ± 10.41 mPa s at 50 s^−1^ and 697.87 ± 7.84 mPa s at 300 s^−1^; IDDSI Level 4) was used for Semisolid trials. A volume of 10 mL was administered using a spoon
3. Mixed consistency 1 (biscuits-with-milk): 10 mL of a mix of room temperature skim milk and biscuits (IDDSI Level 7; fluid: skim milk < 50 mPa·s at 50 and 300 s^−1^, IDDSI Level 0; food: soaked biscuits IDDSI Level 5) was administered using a spoon. The Mixed consistency 1 was prepared using 150 mL of room temperature skim milk and two 8-g dry biscuits divided into four parts each. The biscuits were left to soak for 1 min in the milk before administration
4. Mixed consistency 2 (vegetable-soup): 10 mL of room temperature vegetable soup (Zuppa di Verdura Knorr®, Unilever Italia, Roma) (IDDSI Level 7; fluid IDDSI Level 2 and food IDDSI Level 6) was administered using a spoon
5. Solid: half an 8-g dry biscuit (4 g per trial; IDDSI Level 7) was used for Solid trials

The two different mixed consistencies were selected in order to mimic the typical Italian breakfast and dinner (milk with biscuits and vegetable soup, respectively [8]). For each consistency administered via spoon, at least three consecutive trials were performed. A total of at least 15 boluses were administered under FEES examination. All the subjects received the same instructions during the examination. They were advised to hold the bolus and swallow on command (cued swallow).

FEES examinations were rated independently by three independent operators using the video files; each video was assessed by two independent speech and language therapists (SLTs) using validated ordinal scales for swallowing safety and efficacy; inter-rater reliability between the two raters was analyzed. In case a difference > 1 level at each FEES rating scale occurred between the two raters, a third SLT assessed the videos and decided on both ratings. All the operators had over 5 years of experience in FEES examinations. None of them was involved in the FEES examination and all of them were blind to each other and to participants’ data, since videos were stored in an anonymous form. Consensus scores were used only in cases of disagreement. Three parameters were analyzed using the FEES examination: (1) the location of the head of the bolus at the onset of swallowing; (2) the severity of penetration/aspiration; (3) the amount of pharyngeal residue after the swallow.

- Location of the head of the bolus at the onset of swallowing: similar to the study of Langmore et al. [20] the location of the head of the bolus at the onset of swallowing was scored according to a five points scale. The location score ranges from zero to four, where zero indicates the head of the bolus is behind the tongue; (1) the bolus is at the base of the tongue or valleculae; (2) the head of the bolus moves to lateral channels or the tip of the epiglottis; (3) the head of the bolus is in the piriform sinuses or touches the laryngeal rim on the sides or back; and (4) the head of the bolus falls into the laryngeal vestibule or is aspirated before the swallow [20]. The onset of swallowing was identified as the last endoscopic frame visualizing the bolus before the beginning of the white-out (WO). WO was defined as the first frame of completely white image, it occurs when the mucosa of the pharynx gradually tightens enclosing the tip of the endoscope during pharyngeal muscles contraction and corresponds to the transit of the bolus through the pharynx [6]. The worst bolus head location score for each consistency and for each subject was considered for statistical analyses
- Penetration/aspiration: the severity of penetration/aspiration was rated using the Penetration Aspiration Scale (PAS) [21]. The PAS is an eight-points scale ranging from one (materials do not enter the airway) to eight (materials enter the airway, passes below the vocal folds and no effort is made to eject). Penetration was defined as the bolus entering the laryngeal vestibule over the rim of the larynx (PAS score from two to five). Aspiration was defined as the bolus passing below the true vocal folds (PAS score six or above). The worst PAS score for each consistency and for each subject was considered for statistical analyses
- The amount of pharyngeal residue after the swallow was rated using the Yale Pharyngeal Residue Severity Rating Scale (YPRSRS) vallecula and pyriform sinus [22]. The worst YPRSRS score for each consistency and for each subject was considered for statistical analyses

During the assessment of FEES video recordings, dysphagia pathophysiology was characterized for each patient according to the videoendoscopic classification proposed by Desuter [23]. In particular, the presence of the following six pathophysiological mechanisms was assessed: protective deficit, posterior oral incontinence, delayed pharyngeal phase, oropharyngeal dyspraxia, propulsion deficit, resistive issue.

### Statistical Analysis

Statistical tests were performed using the SPSS 23.0 statistical software (SPSS Inc., Chicago, IL). The Kolmogorov–Smirnov test was used to test the normality of the distribution of FEES parameters among the patients. Since this test demonstrated that the distribution was not normal, non-parametric tests were used. Inter-rater reliability of FEES scoring between the two SLTs was analyzed. Weighted kappa with quadratic weighting was calculated [24]; *k* values were interpreted as follows: ≤ 0.20 poor agreement, 0.21–0.40 fair agreement, 0.41–0.60 moderate, 0.61–0.80 good, and 0.81–1.00 very good [25]. The Kruskal–Wallis with Mann–Whitney post-hoc tests were used to evaluate the differences in the location of the head of the bolus at the onset of swallowing and the amount of food residue left after the swallow according to the different textures. To compare the differences in the frequency of penetration/aspiration among different textures the Fisher exact test was used, since this variable was considered categorical [26, 27]. The significance level was adjusted using the Bonferroni correction at 0.01 in order to avoid type 1 errors by multiple comparisons.

## Results

All the enrolled patients had a total oral intake for at least one consistency and the median FOIS score was five (interquartile range 5–6). All subjects included in the study completed the FEES protocol using five different consistencies without any complications. In all of the patients a white-out period was present and, consequently, it was always possible to identify the time point marking the beginning of each swallow. The time required to complete FEES never exceeded 15 min. FEES scoring inter-rater agreement ranged from good to very good; in particular inter-rater agreement with each of the five different consistencies for the PAS (*k* > 0.85) and for the YPRSRS in the vallecula and pyriform sinus (*k* > 0.80 and k > 0.83, respectively) was very good, while for the location of the head of the bolus was good (*k* > 0.74). The pathophysiology of dysphagia was characterized both in the overall sample and within each diagnostic category (Table 2). The most common pathophysiological mechanism was a delayed pharyngeal phase (75%), followed by posterior oral incontinence (50%) and propulsion deficit (50%). Four (20%) patients showed only one isolated mechanism, ten (50%) patients showed two combined mechanisms, and six (30%) patients showed three combined mechanisms.

**Table 2:** Pathophysiology of dysphagia in the study sample

	Overall *N* = 20	Stroke *N* = 4	Ischemic leukoariosis *N* = 8	Polineuropathy *N* = 3	ALS *N* = 1	Steinert’s disease *N* = 1	Systemic sclerosis *N* = 1	Ataxia *N* = 1	Guillan-Barrè syndrome *N* = 1
Protective deficit	4 (20%)	–	2 (25%)	–	–	1 (100%)	–	1 (100%)	–
Posterior oral incontinence	10 (50%)	4 (100%)	2 (25%)	2 (67%)	1 (100%)	1 (100%)	–	–	–
Delayed pharyngeal phase	15 (75%)	3 (75%)	7 (87.5%)	2 (67%)	1 (100%)	–	1 (100%)	1 (100%)	–
Oropharyngeal dyspraxia	0 (0%)	–	–	–	–	–	–	–	–
Propulsion deficit	10 (50%)	3 (75%)	4 (50%)	1 (33%)	1 (100%)	1 (100%)	–	–	–
Resistive issue	3 (15%)	–	2 (25%)	–	–	–	–	–	1 (100%)

Data are reported as absolute (relative) frequencies

### Location of the Head of the Bolus at the Onset of Swallowing

The most common location of the Liquid and biscuits-with-milk boluses at the onset of swallowing was in the laryngeal vestibule (7 out of 20 patients for both consistencies). The most common location of the Semisolid and vegetable-soup boluses at the onset of swallowing was at the level of the tip of the epiglottis or into the lateral channels (10 out of 20 patients and 8 out of 20 patients, respectively). Finally, the most common location of the Solid bolus at the onset of swallowing was at the level of the base of the tongue or valleculae (10 out of 20 patients). The median location scores obtained using the scale proposed by Langmore et al. [20] are reported in Table 3. The differences in the location scores were found to be significant using the Kruskal–Wallis test (*p* = 0.001). In particular, the location score was significantly higher for Liquid, biscuits-with-milk and vegetable-soup than for the Semisolid and Solid. No difference in the location score was found among the Liquid, biscuits-with-milk and vegetable-soup and between the Semisolid and Solid. The results of the post-hoc analysis are reported in Table 4.

**Table 3:** The results of the FEES examination obtained in the cohort of patients are reported

	Liquid	Semisolid	Solid	Biscuits-with-milk	Vegetable-soup
Location score	2 (1–3)	2 (0.75–2)	1 (1–2)	3 (2–4)	2 (2–3)
YPRSRS vallecula	3 (2–3)	4 (3–4)	4 (2.5–4)	3 (2–3)	3 (2–3.75)
YPRSRS pyriform sinus	2 (2–2)	2 (2–2)	2 (1–2)	2 (1.75–2)	1 (1–2)
PAS					
Normal	4 (20%)	13 (65%)	15 (75%)	5 (25%)	12 (60%)
Penetration	9 (45%)	5 (25%)	5 (25%)	11 (55%)	6 (30%)
Aspiration	7 (35%)	2 (10%)	0 (0%)	4 (20%)	2 (10%)

The results are reported as median and interquartile range (in brackets) for the ordinal data and as frequencies for the categorical data. The PAS scores were categorized in Normal, Penetration and Aspiration

*PAS* Penetration Aspiration Scale, *YPRSRS* Yale Pharyngeal Residue Severity Rating Scale

**Table 4:** Comparison among the location scores obtained with the five different consistencies

	Semisolid	Solid	Biscuits-with-milk	Vegetable-soup
Liquid	0.009*	0.005*	0.196	0.729
Semisolid	–	0.841	0.001*	0.008*
Solid	–	–	0.001*	0.002*
Biscuits-with-milk	–	–	–	0.059
Vegetable-soup	–	–	–	–

The results of post-hoc analysis performed using Mann–Whitney test are reported

*p < 0.01

### Penetration and Aspiration

The differences in the distribution of the three PAS categories (normal, penetration, aspiration) with the five consistencies are reported in Table 3. Penetration was more frequent with biscuits-with-milk (11 out of 20 patients), followed by Liquid (9 patients), vegetable-soup (6 patients), semisolid (5 patients) and solid (5 patients). Aspiration was more frequent with Liquid (7 patients), followed by biscuits-with-milk (4 patients), vegetable-soup (2 patients), and Semisolid (2 patients). No aspiration was demonstrated for the Solid (0 patient). These differences were found to be significant using the Fisher’s exact test (*p* = 0.017). The results of the post-hoc analysis are reported in Table 5. No difference in the distribution of the three PAS categories among Semisolid, Solid, and vegetable-soup on one side and between Liquid and biscuits-with-milk on the other was found. However, significant differences in the distribution of the three PAS categories in the comparison between Liquid vs. Semisolid, Liquid vs. Solid, Liquid vs. vegetable-soup, and biscuits-with-milk vs. Solid were found.

**Table 5:** Comparison among the three PAS categories (normal, penetration, aspiration) obtained with the five different consistencies

	Semisolid	Solid	Biscuits-with-milk	Vegetable-soup
Liquid	0.001*	0.002*	0.270	0.006*
Semisolid	–	0.337	0.039	0.937
Solid	–	–	0.004*	0.298
Biscuits-with-milk	–	–	–	0.081
Vegetable-soup	–	–	–	–

The results of post-hoc analysis performed using Fisher test are reported

*p < 0.01

### Pharyngeal Residue

The results obtained in the YPRSRS vallecula and pyriform sinus are reported in Table 3. No significant differences according to the consistency were demonstrated at Kruskal–Wallis test (*p* = 0.051 and *p* = 0.071 for the YPRSRS vallecula and pyriform sinus respectively).

## Discussion

Combining solid and liquid textures in a single food is common during an ordinary meal [10]. For example, biscuits-with-milk and vegetable soups are frequently consumed during a typical Italian breakfast and dinner, respectively [8]. However, mixed consistencies are rarely tested during FEES and VFSS protocols in dysphagic patients even though previous studies suggested that swallowing mixed consistencies is a qualitatively different task than swallowing single consistencies [9, 10, 12, 14]. In the present study the effect of mixed consistencies on different aspect of swallowing in patients with dysphagia was analyzed using FEES. The results here reported provide a better understanding of patient’s swallowing abilities in real-life situations.

### Location of the Head of the Bolus at the Swallow Onset

A significant difference in the location of the head of the bolus at the swallow onset was demonstrated. In particular, when consuming biscuits-with-milk and Liquid the swallow onset occurred more often when the boluses were located in the laryngeal vestibule, while with the other consistencies the swallow onset occurred more frequently when the boluses were located either in the valleculae (Solid) or at the level of the epiglottis/lateral channels (Semisolid and vegetable-soup). These findings are consistent with previous reports. A review by Steele and colleagues on the influence of food texture and liquid consistency on swallowing physiology highlighted that both studies on healthy subjects and those on patients with dysphagia reported a different location of the bolus at swallow onset with thicker boluses compared with liquids [28]. Saitoh et al. [9] who studied the effect of chewing and bolus consistency on swallowing in a group of 15 healthy subjects reported that in the upright position the position of the bolus at the swallow onset was related to both chewing and bolus consistency. The authors found that at swallow onset the leading edge of the solid bolus was located at the level of the upper oropharynx or valleculae [9]. Similarly, in the present study the Solid bolus was propelled into the pharynx before swallow onset probably because of an active process driven by action of the tongue pressing against the palate [9].

As far as it is concerned the mixed and liquid consistencies, the early movement of the Liquid, biscuits-with-milk and vegetable-soup into the hypopharynx found in the present study is similar to previous reports. Saitoh et al. [9] found that the leading edge of the bolus was frequently located at the level of the hypopharynx when the non-dyspahgic subjects swallowed mixed consistency (corned beef mixed with liquid barium) or chewed liquid. Yamada et al. [11] reported that the location of the head of the bolus in the pharynx at the swallow onset was related to the consistency of the mixed food and found that with thicker two-phase food the swallow is not elicited immediately when the liquid component reaches the pharynx. Humbert et al. [12] found that healthy subjects often allowed thin liquids to flow into the valleculae or pyriform sinuses while masticating the solid part of the bolus. In addition, Lee et al. [7] found that the pharyngeal swallow of the mixed and liquid consistencies in dysphagic patients usually started when the head of the bolus was located in the hypopharynx. Kang et al. [14] found that with a mixed consistency bolus, posterior premature spillage was observed in almost half of patients leading to penetration or aspiration in some of them. However, it must be noted that the bolus position alone does not differentiate between normal and pathologic swallowing since also in non-dysphagic subjects the swallow of liquids might initiate when the bolus head is well below the faucial pillars without associated penetration or aspiration [9, 12, 29]. The premature spillage of the liquid and mixed consistencies appears to be mainly related to gravity [9] and might be related to the fact that the soft palate seal to the back of the tongue is broken by active tongue movement during the chewing [9, 30] or by an oropharyngeal weakness caused by the disease that finally lead to dysphagia. This latter hypothesis is consistent with the results of Lee et al. [7] and might explain why, in the cohort of patients in the present study, onset of the pharyngeal swallow with the Liquid bolus was located more frequently at the level of the laryngeal vestibule. Along with the effects of the bolus itself on swallowing physiology, the majority of the patients in the present study exhibited a delayed pharyngeal response and a posterior oral incontinence, typically leading to a higher frequency of premature spillage of the bolus in the pharynx. Thus, the pathophysiological mechanisms of dysphagia in the recruited sample may have exacerbated the impact of liquids and foods consistency on the location of the head of the bolus at swallow onset, also in a cued condition, as in this condition it is unusual to see the head of the bolus reaching the piriform sinuses [31, 32].

### Penetration and Aspiration

The FEES examination demonstrated that penetration was more frequent with biscuits-with-milk, while aspiration was more frequent with Liquid, followed by biscuits-with-milk and vegetable-soup, Semisolid and Solid. In particular, no differences in the distribution of the three PAS categories between Liquids and biscuits-with-milk were found as well as among vegetable-soup, Semisolid and Solid. These data suggest that the viscosity of the ingested food significantly affect the safety of deglutition. This finding is similar to those reported by Clavé et al. [33] who found that in patients with neurogenic dysphagia, increasing viscosity significantly improved efficacy of deglutition and brought about a dramatic improvement on safety by minimizing penetration and aspiration during swallow [33]. Interestingly, the risk of penetration-aspiration for biscuits-with-milk and Liquid is similar, while vegetable-soup safer, as well as Solid and Semisolid. Only few data are available in the international literature about the risk of penetration-aspiration with mixed consistencies and consequently it appears very difficult to compare the results here reported. In addition, in the majority of previous studies only one mixed consistency was tested. Ozaki et al. [10] who analysed the risk of penetration/aspiration with different consistencies in a large group of dysphagic patients using VFSS reported that the risk of aspiration was higher for the mixed consistency, while the risk of penetration was higher for the liquid consistency. Also, Kang et al. [14] reported penetration with mixed consistency in 6 patients in a group of 49 patients without abnormal findings with liquids. On the contrary, Lee et al. [7] found that the risk of aspiration was higher for the liquid consistency, while the risk of penetration was higher for the mixed consistency. These inconsistencies may be related to several causes. First of all, in the analyses of Ozaki et al. and Kang et al. [10, 14] the volume of the tested consistencies was different as well as the method of delivery (a syringe for liquid, in the usual patient’s manner for solid and mixed consistencies). In the present study all the consistencies, with the only exception for the solid one, were delivered using a spoon of 10 mL thus assuring equivalence of volume and delivery method. Second, in the study of Ozaki et al. [10] the penetration/aspirations events occurred during each food trial were evaluated by agreement of at least two researches including physiatrists, dentist and/or speech therapists. In the present study the severity of penetration/aspiration was evaluated using a scale with known reliability and clinical validity (the PAS scale [21]). In addition, each video recording was rated by two independent operators with over 5 years of experience in FEES examinations. Finally, the texture and the rheology of the mixed consistencies used in the present study and in the ones of Ozaki et al. and Kang et al. [10, 14] were different. Lee et al. [7] suggested that the texture of a mixed consistency could affect the level of stimulation of the sensory afferent pathway thus modulating the sensorimotor feedback which is important to protect the airway [7, 34]. Both biscuits-with-milk and vegetable soup have a little rough surface area and can be divided more evenly with liquid, consequently they could be more stimulative compared with corned beef with liquid barium [7]. Moreover, also the rheology of food might have played a role, with thicker mixed consistencies (such as the vegetable soup) forming a more cohesive bolus which was less likely to result in a penetration-aspiration event. This hypothesis might explain why the frequency of penetration and aspiration was lower with vegetable-soup than with biscuits-with-milk even if this difference was found not to be significant at Fisher exact test.

### Pharyngeal Residue

The differences in the severity of pharyngeal residue among the various consistencies were found not significant in the present study. Nonetheless, the results here reported suggest that the residue increases with thicker consistencies (Semisolid and Solid) both in the valleculae and in the pyriform sinuses. These results are similar to those reported by Lee et al. [7] and in accordance with the findings of the review by Steele et al. [28]. Two aspects may have contributed to the findings. The first one is related to the food consistency itself, as residue is increased with increasing viscosity also in healthy adults. The second one is that half of the patients exhibited a propulsive deficit, related to poor pharyngeal contraction and tongue-base retraction, that provoke more residues as the effort required for swallowing increases with thicker and harder foods [28].

### Study Limitations

This study has several limitations. First of all, the number of enrolled patients is limited. Consequently, the results here reported should be considered as preliminary and a larger dysphagic population is needed in order to confirm these findings. Second, the cohort of patients is not homogeneous since dysphagia was related to different causes and different pathophysiological mechanisms. Third, the use of FEES instead of VFSS did not allow to perform temporal measures, such as the pharyngeal delay time, but had the advantage to allow patients to eat real food and take typical bolus sizes. In particular, potential pharyngeal swallow delay could not be evaluated using temporal measures. Instead, the following criteria were adopted: (1) the patients were able to keep the bolus in the mouth; (2) when the patients were asked to swallow, the head of the bolus flowed beyond the glossopharyngeal ligaments and a delay of glottis closure or laryngeal elevation or tongue propulsion was visible [23]. Future studies are needed using temporal measures to analyze the impact of different pathophysiological mechanisms on penetration/aspiration and residue. Fourth, randomization of bolus presentation was not performed even if it would have allowed to control for an order effect. By providing the different consistencies with a random order the results would have been more consistent. Fifth, the intra-rater reliability was not evaluated. Data on intra-rater reliability would have further strengthened the reliability of the FEES examination. Finally, the application of a cued swallow protocol does not allow to translate the results here reported to everyday meals consumption. Previous videofluoroscopic studies comparing cued versus non-cued swallows showed that an advanced position of the bolus at swallow onset is observed significantly less frequently in the context of a cued swallow [31, 32]; it is therefore possible that using a non-cued paradigm, data on penetration and aspiration and those on the location of the head of the bolus at the onset of swallowing would show significantly worse scores with the different consistencies; further studies are necessary to analyze the impact of mixed consistencies using a non-cued protocol.

## Conclusion

In conclusion, clinicians should be aware that the pattern of food transport and swallowing is different among different consistencies and consequently it might be advisable to include also mixed consistencies in the evaluation of individuals with dysphagia. As far as it is concerned the safety of deglutition, swallowing of a thin mixed consistency, such as biscuits-with-milk does not seem to be more dangerous than swallowing liquids, while the consumption of a thicker mixed consistency, such as vegetable soups, appears safer. When considering the location of the head of the bolus at the swallow onset and the risk of penetration-aspiration, in fact, the deglutition of a thicker mixed consistency appears similar to a semisolid food. The “a priori” exclusion of mixed consistencies from the regular diet of a dysphagic patient might be avoided, rather the texture and the rheology of the mixed food should be decided according to the severity and the pathophysiology of the swallowing disorder.
